# Supplementary material for: Toward total recall: Enhancing data FAIRness through AI-driven metadata standardization
Source: Gigascience. 2026 Mar 3;15:giag019. doi: 10.1093/gigascience/giag019 (PMC13108262; doi:10.1093/gigascience/giag019)
Supplement: giag019_Authors_Response_To_Reviewer_Comments_original_submission [file giag019_authors_response_to_reviewer_comments_original_submission.pdf]

# Author's Response To Reviewer Comments

We sincerely thank the reviewers for their thorough evaluation and constructive feedback. We have carefully addressed each comment, and a summary of our revisions is provided below

----------------------------------------------------------------------------------------------------------

Reviewer #1: Thank you for your detailed comments. We address your primary concerns below.

1. Concerns on query complexity:

"The validation conducted appears to be based on relatively simple queries focused on specific cancer types and the "tissue" field. To robustly demonstrate the generalizability and efficacy of the proposed method, it would be highly beneficial to broaden the scope and complexity of the queries."

We understand the concerns about the extent of query experimentation. Our goal was to highlight how existing systems struggle with even simple queries, which is the mechanism on which most search indexers rely, and to show how recall improves under our approach. We have experimented with more complex queries and we do see improvements in recall. However, the complexity of the query adds more noise to the results, and describing those results reduces the clarity of the overall presentation. We find that the fact that dataset search is failing on the most basic queries—the kind of queries that take place in the real world—to be much more alarming than the problem with more complex queries. We have added a discussion of this issue in the penultimate paragraph in the Methods section.

2. Concerns on database variety:

"Given the quality expected from a journal like GigaScience, the current validation on subsets of BioSample and GEO, while a good start, might not be extensive enough to fully verify the method's reliability and uncover its potential limitations across diverse biological domains. Incorporating a wider variety of datasets from different scientific disciplines or from other major public repositories would significantly bolster the claims of reliability and generalizability."

We have demonstrated improved recall across three cohorts drawn from two databases that differ markedly from one another. Since high-quality metadata for direct comparison are unavailable, each database and cohort requires the time-consuming development of a gold standard for recall computation. Given the lack of well specified metadata templates (as is typical in the real world, both BioSample and GEO specify only “principles” and data types at best, not specific field name–value restriction pairs), our in-house metadata engineering team built a CEDAR template to the best of their ability. After constructing the template, we worked with domain experts to develop a gold standard template through approximation methods suitable for each domain. Since search and corresponding effects on recall have not yet been widely benchmarked, we have painstakingly constructed these two benchmark datasets from scratch. If there are many off-the-shelf datasets curated for scientific search, we could easily incorporate them. As we need to extend existing databases into gold standards for search, we posit that using two diverse databases such as BioSample and GEO provide a good starting point to establish that making datasets FAIR improves recall. Based on these results, we are confident that the observed trend of improved recall will scale to additional databases, and that further computation is desirable, but not necessary. For example, the recent 2025 Gigascience article [1] uses only one database, namely BioSample.

3. Comparison of RAG and CEDAR templates:

"The use of Retrieval-Augmented Generation paradigms to improve LLM capabilities is becoming increasingly common in various NLP applications. While the specific application to metadata standardization with CEDAR templates is valuable, the manuscript could benefit from a more explicit discussion of how its "template-augmented prompt engineering" (as described in the discussion) distinguishes itself from or contributes uniquely to the broader RAG landscape."

We have highlighted the benefits of CEDAR templates, including adherence to community standards, the provision of guardrails for the LLM generation process, and support for template authorship. At the surface, a RAG system can improve recall. However, this method would involve the overhaul of many search indexers that exist on a plethora of databases already. Hence, we find our analysis closer to the real-world scenario.. We have added text regarding this issue in the discussion, paragraph 3. We have also demonstrated the advantages of using these templates in combination with RAG systems, particularly in the context of query complexity, namely that information retrieval is enhanced with or without RAG.

4. Concerns on figure and table clarity
"Ensure all figures and tables are clearly labelled, easily readable, and self-explanatory."

We have re-written all the captions with more clarity

5. Request for prompting engineering details

"Consider providing more details on the prompt engineering strategies used for GPT-4 to allow for better reproducibility."

We have added the prompts in Appendix A

---------------------------------------------------------------------------------------------------

Reviewer #2: Thank you for your detailed and positive feedback. We have incorporated your minor suggestions and edits, which have greatly improved the readability of the paper.

---------------------------------------------------------------------------------------------------

Reviewer #3: Thank you for your detailed comments. We address your primary concerns below.

1. Concerns regarding Zenodo data:

"The dataset shared on Zenodo is insufficiently documented. The short description ("The repository contains records from…") and the file names are not descriptive enough to allow readers to easily identify the content of each file. Please expand the description (e.g., using bullet points or a table) clearly explaining what each file contains and how it relates to the analyses in the paper."

We have revised the dataset and accompanying documentation to ensure it is more descriptive and self-contained. We have also added the numerical analyses in a spreadsheet in Zenodo.

2. Concerns regarding metric completeness:

"Table 3 describes the three evaluation metrics (precision, recall, F1-score), but the definitions of true positives, false positives, and false negatives in the specific context of this study are not provided anywhere in the manuscript. Please add explicit explanations with concrete examples of what counts as TP, FP, TN, and FN in your metadata curation framework. Please consider adding a simplified table (perhaps as Table 4) to illustrate your classification logic."

We have added the definitions of the analyses, including TP, FP, TN, and FN values, and improved the clarity of column labels. Additional LLM-related information has also been included in the appendix B. [Table 4, page 5].

3. Concerns regarding figure clarity:

"Please carefully review the values and figure numbering in Paragraph. For example, you state "baseline metadata to 44% with GPT-4 guided by the data dictionary" (Line 6, Paragraph 1), however in Figure 5 this

value appears closer to ~21%. Same applies to multiple values listed in the paragraph. Also, current references to Figures 7 and 8 seem to correspond to Figures 6 and 7 respectively."

We have resolved the ambiguities in both the figures and their corresponding text. We apologize for the confusion caused by accidentally skipping a figure. [Page 6]

4. Concerns regarding statistical significance:

"Paired t-test: ANOVA would be more appropriate than multiple paired t-tests to avoid the inflations of errors. Furthermore, please provide detailed statistical output (not just p <0.01), such as a table with SS, df, MS, F statistics and p values."

We have incorporated the requested statistical analyses, including ANOVA values in Appendix C.

5. Request for fine-grained results for sub-cohorts and other LLMs:

"In paragraph 4, you present dataset-specific results (liver, lung, ovarian) in text only. Please provide figures (similar to Figure 7) for each category, ideally in supplementary materials. In paragraph 5, you discuss results for LLaMA-3, MedLLaMA-2, and Mistral without visual support. Consider including these models in Figures 5-7 or presenting additional figures in the supplementary materials."

These results have been added to the appendix B.

6. Request to expand abbreviations in the abstract

This request has been implemented.

We would like to take this opportunity to thank the reviewers for their time and their detailed comments that have helped us polish the manuscript to a better quality.

------------------------------------------------------------

References:

[1] Shuya Ikeda, Zhaonan Zou, Hidemasa Bono, Yuki Moriya, Shuichi Kawashima, Toshiaki Katayama, Shinya Oki, Tazro Ohta, Extraction of biological terms using large language models enhances the usability of metadata in the BioSample database, GigaScience, Volume 14, 2025, giaf070, https://doi.org/10.1093/gigascience/giaf070

Editorial Manager®

**Cookie Preference Center**

We use cookies which are necessary to make our site work. We may also use additional cookies to analyze, improve and personalize our content and your digital experience. For more information, see our Cookie Policy.

You may choose not to allow some types of cookies. However, blocking some types may impact your experience of our site and the services we are able to offer. See the different category headings below to find out more or change your settings.

Allow all

## Manage Consent Preferences

#### Strictly Necessary Cookies

**Always active**

These cookies are necessary for the website to function and cannot be switched off in our systems. They are usually only set in response to actions made by you which amount to a request for services, such as setting your privacy preferences, logging in or filling in forms. You can set your browser to block or alert you about these cookies, but some parts of the site will not then work.

#### Functional Cookies

These cookies enable the website to provide enhanced functionality and personalisation. They may be set by us or by third party providers whose services we have added to our pages. If you do not allow these cookies then some or all of these services may not function properly.

#### Performance Cookies

These cookies allow us to count visits and traffic sources so we can measure and improve the performance of our site. They help us to know which pages are the most and least popular and see how visitors move around the site.

#### Targeting Cookies

These cookies may be set through our site by our advertising partners. They may be used by those companies to build a profile of your interests and show you relevant adverts on other sites. If you do not allow these cookies, you will experience less targeted advertising.

### Cookie List

Clear

Apply Cancel

Consent Leg.Interest

Confirm my choices

Power[illegible]
