# Supplementary material for: Toward total recall: Enhancing data FAIRness through AI-driven metadata standardization
Source: Gigascience. 2026 Mar 3;15:giag019. doi: 10.1093/gigascience/giag019 (PMC13108262; doi:10.1093/gigascience/giag019)
Supplement: giag019_Reviewer_2_Report_orignal_submission — Reviewer 2 -- 8/9/2025 [file giag019_reviewer_2_report_orignal_submission.pdf]

Reviewer Report

Title: Toward Total Recall: Enhancing Data FAIRness through AI-Driven Metadata Standardization

Version: Original Submission **Date:** 8/9/2025

Reviewer name: Reviewer 2

Reviewer Comments to Author:

In my opinion, this paper is appropriate for publication and usefully adds to the scientific literature. Indeed I will refer to this paper as an exempler in least one current work. There are some minor corrections and clarifications needed though.1) Are the methods appropriate to the aims of the study, are they well described, and are necessary controls included?The methods are well described and appropriate.Improvements are needed around the "approximate gold standard" description aspects though.2) Are the conclusions adequately supported by the data shown?Yes3) Please indicate the quality of language in the manuscript. Does it require a heavy editing for language and clarity?The standard of English is excellent and clear throughout. A slight improvement would be to reduce the reading age by decreasing the length of some of the longer sentences. That is not necessary as all were readily parseable on a first read.4) Are you able to assess all statistics in the manuscript, including the appropriateness of statistical tests used?Yes. The statistical test are descrived and the input and statistical output are accessible.Output data is available in Zenodo.Minor suggested corrections/enhancements:â€”â€”â€”â€”â€”â€”â€”â€”â€”â€”â€”â€”â€”â€”â€”â€”â€”â€”â€”-----5) P2 paragraph 1 "Scientific metadata consists of pairs of" to "Scientific metadata usually consists of pairs of"The reason is that "description" or "comment" can contain a plethora of metadata, as acknowledged elsewhere in your paper.6) P2 paragraph 1: Biosamples are used to annotate many samples outwith those of biomedical studies, that is not clear in what you write. For example there are many seawater, freshwater and non-human/pathogen samples. Yes your query is only of human samples.7) P2 paragraph 2: when describing ontologies, it would be useful to outline one or two advantages over flat controlled vocabularies. e.g. allow different levels of granularity.8) P3: Figure 2: what is the meaning of %? The proportion of eixsting human biosample records that contain a tissue in Uberon using this value?9) P4 paragraph 2. P4 Table2 + paragraph3:Generating the "approximate gold standard". Is it likely that the recall and precision has been improved over baseline, by the baseline not having any basic linguistics stemming etc. done which the LLMs have learnt to do and most doing manual or API calls would consider? IMHO it would be useful to address or acknowledge this possibilit in either the method or the discussion.The phrase "approximate gold standard" is amusing.Question: what proportion of your "approximate gold standard" did you examine manually? Without knowing this, the precision is less firmly based. (Yes, in the paper you did say something about wanting it automated to enable the scale etc.)10) P5/6 where is Figure 8? It was missing my PDF. The data behind the figure 8 is described in the text P5 paragraph2.11) P5 column2 paragraph 2. The "difficulties" is coming across as if it was a quick try. It would be better to rephrase this to ackowledge the effort your team will have probably put in. (Others including Dagdelen et al. 2024 [your #13 reference] and from personal experience, have successfully used Llama2, although they have mainly been on blocks of text rather than a structured

form.)P7 col1 paragraph 4. "The corrected database" may be better as something like the "The enhanced database" . Sorry, for being pedantic here. Your methodology will incorrectly assign/change a small percentage of the valid values.And finally several comments: (Entirely up to you whether to address these or not.)12) P5/7 results or in discussion - for the BioSample did you descriminate between those that were sequence (e.g. in INSDC) and those that were something else e.g. image (a minority may be multiple omics and image etc.)? GEO will be sequence or array.13) FYI: For the BioSample records that originate from the INSDC, most of the important field_names are GSC MIxS standard terms. The values are currently only validated for some field_names, hence the varying quality. The INSDC members manage archives and most metadata changes to record require author permission. Having a more standardised AI ready version of biosamples as you describe, would be useful for the scientific community.14) In your demonstration you had focused on some useful biomedical field_names. It would be interesting applying this to other sample types in BioSamples e.g. environmental ones. Also how well does the LLM+CEDAR work with fields that are not a "textual" string e.g. age(like in your example), dates, lat/lon coordinates, boolean etc. How well did the LLM+CEDAR clean up the sex field? (which is well know to be annotated in an inventive number of ways)

Level of Interest

Please indicate how interesting you found the manuscript: Choose an item.

Quality of Written English

Please indicate the quality of language in the manuscript: Choose an item.

Declaration of Competing Interests

Please complete a declaration of competing interests, considering the following questions:

- Have you in the past five years received reimbursements, fees, funding, or salary from an organisation that may in any way gain or lose financially from the publication of this manuscript, either now or in the future?
- Do you hold any stocks or shares in an organisation that may in any way gain or lose financially from the publication of this manuscript, either now or in the future?
- Do you hold or are you currently applying for any patents relating to the content of the manuscript?
- Have you received reimbursements, fees, funding, or salary from an organization that holds or has applied for patents relating to the content of the manuscript?
- Do you have any other financial competing interests?
- Do you have any non-financial competing interests in relation to this paper?

If you can answer no to all of the above, write 'I declare that I have no competing interests' below. If your reply is yes to any, please give details below.

I declare that I have no competing interests.

I agree to the open peer review policy of the journal. I understand that my name will be included on my report to the authors and, if the manuscript is accepted for publication, my named report including any attachments I upload will be posted on the website along with the authors' responses. I agree for my report to be made available under an Open Access Creative Commons CC-BY license (http://creativecommons.org/licenses/by/4.0/). I understand that any comments which I do not wish to be included in my named report can be included as confidential comments to the editors, which will not be published.

Choose an item.

To further support our reviewers, we have joined with Publons, where you can gain additional credit to further highlight your hard work (see: https://publons.com/journal/530/gigascience). On publication of this paper, your review will be automatically added to Publons, you can then choose whether or not to claim your Publons credit. I understand this statement.

Yes Choose an item.
