# Supplementary material for: Toward total recall: Enhancing data FAIRness through AI-driven metadata standardization
Source: Gigascience. 2026 Mar 3;15:giag019. doi: 10.1093/gigascience/giag019 (PMC13108262; doi:10.1093/gigascience/giag019)
Supplement: giag019_Reviewer_3_Report_original_submission — Reviewer 3 -- 8/15/2025 [file giag019_reviewer_3_report_original_submission.pdf]

Reviewer Report

Title: Toward Total Recall: Enhancing Data FAIRness through AI-Driven Metadata Standardization

Version: Original Submission **Date:** 8/15/2025

Reviewer name: Reviewer 3

Reviewer Comments to Author:

This study addresses a major challenge in implementing FAIR data practices - namely, the incompleteness, inconsistency, and inaccuracy of metadata. The AI-assisted approach demonstrated here offers significant value across scientific disciplines and is particularly timely given the increasing emphasis on "AI readiness" of data. This is long-needed work, and the manuscript is very well written and engaging to read. However, several areas require clarification and improvement before the paper is ready for publication. Please see my comments below.Data Availability (Zenodo): The dataset shared on Zenodo is insufficiently documented. The short description ("The repository contains records from…") and the file names are not descriptive enough to allow readers to easily identify the content of each file. Please expand the description (e.g., using bullet points or a table) clearly explaining what each file contains and how it relates to the analyses in the paper.Methods:- Table 3 describes the three evaluation metrics (precision, recall, F1-score), but the definitions of true positives, false positives, and false negatives in the specific context of this study are not provided anywhere in the manuscript. Please add explicit explanations with concrete examples of what counts as TP, FP, TN, and FN in your metadata curation framework. Please consider adding a simplified table (perhaps as Table 4) to illustrate your classification logic. For example, possible columns might include:o "Query value"o "Ground truth value"o "Curated by GPT-4 + DD"o "Curated by GPT-4 + CEDAR"o "Curated by LLaMA-3"o "Curated by MedLLaMA-2"- Each cell could show the predicted value (e.g., "Lung," "Liver," "Blood," "Unknown") along with a symbol indicating TP, FP, TN, or FN.- Including this type of table for all 4,800 records in the Zenodo dataset would also greatly improve reproducibility.- Please note that the above suggested table structure is based on my current understanding of the study methods. However as I said, the methods are not clearly described in the current manuscript so I may be misunderstanding it all and if that's the case, this table structure may not make sense. As it stands, the methods section does not provide enough detail to confirm whether precision, recall, and F1-score are applied appropriately. Without clearer definitions and examples as above, it is difficult to assess whether the formulas are valid for your study design.Results:- Please carefully review the values and figure numbering in Paragraph. For example, you state "baseline metadata to 44% with GPT-4 guided by the data dictionary" (Line 6, Paragraph 1), however in Figure 5 this value appears closer to ~21%. Same applies to multiple values listed in the paragraph. Also, current references to Figures 7 and 8 seem to correspond to Figures 6 and 7 respectively.- Paired t-test: ANOVA would be more appropriate than multiple paired t-tests to avoid the inflations of errors. Furthermore, please detailed statistical output (not just $p < 0.01$), such as a table with SS, df, MS, F statistics and p values.- In paragraph 4, you present dataset-specific results (liver, lung, ovarian) in text only. Please provide figures (similar to Figure 7) for each category, ideally in supplementary materials.- In paragraph 5, you discuss results for LLaMA-3, MedLLaMA-2, and Mistral

without visual support. Consider including these models in Figures 5-7 or presenting additional figures in the supplementary materials.Minor comments:- Ensure all abbreviations are expanded at first mention, including in the abstract (e.g., CEDAR, GEO, PBMC).Overall, this is an important and well-executed study with strong potential impact. However improvements are required to provide clearer and more detailed descriptions in methods and results presentations to strengthen the manuscript and improve its reproducibility.

Level of Interest

Please indicate how interesting you found the manuscript: Choose an item.

Quality of Written English

Please indicate the quality of language in the manuscript: Choose an item.

Declaration of Competing Interests

Please complete a declaration of competing interests, considering the following questions:

- Have you in the past five years received reimbursements, fees, funding, or salary from an organisation that may in any way gain or lose financially from the publication of this manuscript, either now or in the future?
- Do you hold any stocks or shares in an organisation that may in any way gain or lose financially from the publication of this manuscript, either now or in the future?
- Do you hold or are you currently applying for any patents relating to the content of the manuscript?
- Have you received reimbursements, fees, funding, or salary from an organization that holds or has applied for patents relating to the content of the manuscript?
- Do you have any other financial competing interests?
- Do you have any non-financial competing interests in relation to this paper?

If you can answer no to all of the above, write 'I declare that I have no competing interests' below. If your reply is yes to any, please give details below.

I declare that I have no competing interests.

I agree to the open peer review policy of the journal. I understand that my name will be included on my report to the authors and, if the manuscript is accepted for publication, my named report including any attachments I upload will be posted on the website along with the authors' responses. I agree for my report to be made available under an Open Access Creative Commons CC-BY license (http://creativecommons.org/licenses/by/4.0/). I understand that any comments which I do not wish to

be included in my named report can be included as confidential comments to the editors, which will not be published.

Choose an item.

To further support our reviewers, we have joined with Publons, where you can gain additional credit to further highlight your hard work (see: https://publons.com/journal/530/gigascience). On publication of this paper, your review will be automatically added to Publons, you can then choose whether or not to claim your Publons credit. I understand this statement.

Yes Choose an item.
